# Furin Is the Major Proprotein Convertase Required for KISS1-to-Kisspeptin Processing

**DOI:** 10.1371/journal.pone.0084958

**Published:** 2014-01-13

**Authors:** Sitaram Harihar, Keke M. Pounds, Tomoo Iwakuma, Nabil G. Seidah, Danny R. Welch

**Affiliations:** 1 Department of Cancer Biology, The University of Kansas Medical Center, Kansas City, Kansas, United States of America; 2 The University of Kansas Cancer Center, The University of Kansas Medical Center, Kansas City, Kansas, United States of America; 3 Department of Molecular Physiology, The University of Kansas Medical Center, Kansas City, Kansas, United States of America; 4 Department of Pathology and Laboratory Medicine, The University of Kansas Medical Center, Kansas City, Kansas, United States of America; 5 Clinical Research Institute of Montreal, affiliated to Université de Montréal, Montréal, Québec, Canada; University of South Alabama, United States of America

## Abstract

KISS1 is a broadly functional secreted proprotein that is then processed into small peptides, termed kisspeptins (KP). Since sequence analysis showed cleavage at KR or RR dibasic sites of the nascent protein, it was hypothesized that enzyme(s) belonging to the proprotein convertase family of proteases process KISS1 to generate KP. To this end, cell lines over-expressing KISS1 were treated with the proprotein convertase inhibitors, Dec-RVKR-CMK and α1-PDX, and KISS1 processing was completely inhibited. To identify the specific enzyme(s) responsible for KISS1 processing, mRNA expression was systematically analyzed for six proprotein convertases found in secretory pathways. Consistent expression of the three proteases – furin, PCSK5 and PCSK7 – were potentially implicated in KISS1 processing. However, shRNA-mediated knockdown of furin – but not PCSK5 or PCSK7 – blocked KISS1 processing. Thus, furin appears to be the essential enzyme for the generation of kisspeptins.

## Introduction

A family of metastasis suppressors has emerged as key regulators of metastasis. They act on multiple tumor types by altering one or more steps in the metastatic cascade without blocking primary tumor formation [1]. Originally discovered as a metastasis suppressor, KISS1 has since been defined as a neurotransmittor and regulator of diverse cellular functions (reviewed in [2]–[4]) and has been implicated in pathologies such as hypogonadism [2], [3], [5] and Alzheimer's disease [6]. Several laboratories are actively developing therapies based upon KISS1 biology.

The *KISS1* gene and its paralog (*KISS2*) are generally well conserved in diverse species, including teleosts [7]. Comparison of synteny reveals that *KISS* was duplicated before divergence of sarcopterygians and actinopterygians, but that the *KISS2* paralog is lost in placental mammals [8]. Nonetheless, KISS::KISS1R interactions and processing of nascent protein into KP appear to be relatively well conserved across species.

KISS1 protein comprises of 145 amino acids with an N-terminal secretion signal peptide [9], [10]. KISS1 secretion is followed by proteolytic cleavage into KP. Cleavage of a peptide from KISS1 (R^67^-R^124^) followed by amidation results in a 54 amino acid polypeptide, KP54 (G^68^-F^121^). KP54 binds to the KISS1 receptor (KISS1R, formerly known as GPR54, AXOR12), a G_q11_ G-protein coupled receptor, but can then be further cleaved into smaller KP representing the C-terminal 14, 13 or 10 amino acids [11], [12]. The LRF-NH_2_ sequence at the C-terminus of KP14, KP13 and KP10 are critical for KISS1R binding [13], [14].

We previously showed that KISS1 is secreted by tumor cells and this property is critical for its role in metastasis suppression [15]. Further, processing into KP occurs outside of the cell. Polypeptide ends are consistent with cleavage at dibasic residues similar to that observed in neuropeptides, hormones, receptors and viral glycoproteins by the class of enzymes called proprotein convertases (PC) [16]–[18]. In mammals, nine PC are known. The seven human PC that specifically cleave dibasic residues are denoted PCSK1 to PCSK7 (PCSK3 is more commonly known as furin) and cleave their target precursor proteins at specific single or paired basic consensus motif (R/K)X_n_(R/K), where X can be any amino acid except Cys or Pro and n = 0, 2, 4 or 6 amino acids [16].

Furin, PCSK5, PCSK6 and PCSK7 are ubiquitously expressed, widely distributed and contribute to processing of their targets in secretory pathways, cell surface and/or extracellular matrices [19]. Despite variable degrees of redundancy in substrate specificity and function, most experiments reveal that each PC has distinct targets and fulfills specific functions. Knockout mice of individual PC demonstrate that PC are critical for embryogenesis and development. *Furin* ^-/-^, *Pcsk5* ^-/-^ or *Ski-1/S1p* ^-/-^ mice exhibit early embryonic lethality; whereas, *Pcsk6* ^-/-^ mice survive to adulthood, but show defects in axis development as well as craniofacial and cardiac malformations [20]. The importance of several PCs can be underscored by their involvement in multiple human pathological conditions ranging from obesity, viral infections, bacterial infections, Alzheimer's disease, atherosclerosis and cancer (reviewed in [16], [17], [19]).

Despite involvement of KISS1 and KP in so many biological and pathological processes, the enzyme(s) responsible for generation of KP have not yet been defined. This deficiency in knowledge assumes great significance since identifying and modulating these enzymes may play crucial roles in mediating responses to KISS1 and, importantly, understanding of diseases in which these molecules are involved. Based upon the dibasic cleavage predictions, the hypothesis was that KP generation is due to proteolytic processing by one or more members of the proprotein convertase family of proteases.

## Experimental Procedures

### Ethics statement

All animal studies were carried out in strict accordance with the recommendations in the Guide for the Care and Use of Laboratory Animals of the National Institutes of Health. The protocol was approved by University of Kansas Medical Center Institutional Animal Care and Use Committee (Protocol #2011-2011).

Animals were sacrificed by cervical dislocation following anesthesia (ketamine/xylazine) and all efforts were made to minimize suffering.

### Cell lines and cell culture

Human cell lines originating from melanoma (C8161.9; MelJuSo) [21], [22]; breast carcinoma (MDA-MB-231; MDA-MB-435) [23], [24]; cervical carcinoma (HeLa), colorectal carcinoma (LoVo); embryonic kidney (HEK293) and monkey fibroblast (COS7) were either stably transfected with vectors encoding full-length KISS1 cDNA or a cDNA in which KISS1 with an internal FLAG epitope was encoded [15]. Lentiviral vector or pcDNA3 vectors were used for stable expression while adenoviral vectors were used for transient expression experiments (Life Technologies, Carlsbad, CA).

Most cell lines were grown in a 1∶1 mixture of Dulbecco's-modified minimum Eagle's medium and Ham's F-12 medium (DMEM/F-12), supplemented with 5% fetal bovine serum (Life Technologies). LoVo, COS7 and HEK293 cells were grown in DMEM/high glucose media supplemented with 10% FBS (Life Technologies).

All cells were cultured without antibiotics unless under selection pressure. All cells were negative when tested for *Mycoplasma spp.* contamination (Takara-Clontech, Mountain View, CA).

### Detection of KISS1 and KP

To check for KISS1 and KP, the desired cells were plated to near confluence onto 10 cm tissue culture plates; the culture medium was removed and replaced with serum-free medium and incubated for an additional 48 hr. Conditioned medium (CM) was collected and centrifuged at ∼1600 x g (Sorval Legend XTR) for 5 min at 4°C. Cells were pelleted and lysed with RIPA buffer containing protease and phosphatase inhibitors (Catalog #1861281; Pierce, Rockford, IL). Secreted KISS1 and/or KP were immunoprecipitated from 1 ml of CM with an antibody raised against KP54 fragment of KISS1 combined with protein A/G beads and incubated overnight at 4°C [25]. The immunoprecipitated samples were then separated on a 16.5% Tris-tricine peptide gel, transferred onto PVDF and probed with anti-KP54 antibody. For mass spectroscopy, gels were stained with Coomassie blue, and visible bands were excised, digested with either trypsin or Glu-C proteases and identified by matrix-assisted laser desorption/ionization-time of flight mass spectroscopy (MALDI-TOF) and/or electrospray ionization mass spectroscopy/mass spectroscopy (ESI-MS/MS). Partial overlapping sequences derived from digested KP bands were aligned. For analysis of KISS1 processing following treatment with PC inhibitors, cells were incubated with Dec-RVKR-CMK (100 µM) for 24 hr or transduced with adenoviral vector expressing α1-PDX before proceeding with the immunoprecipitation and KISS1/KP detection.

### Taqman quantitative real-time PCR

mRNA levels of KISS1 and six members of the PC family were quantified using real-time PCR analysis (Taqman, Life Technologies) using ABI Viia 7 detection system. Endogenous actin was used as an internal reference. Each sample was assayed in triplicate.

### Western blotting and antibodies

Cells were lysed in RIPA buffer containing a protease and phosphatase inhibitor cocktail (Pierce, Rockford, IL). Protein concentration was determined using a BCA assay. Protein (50 µg) was denatured with Laemmli's buffer at 95°C for 5 min before separation using 4–20% gradient SDS-PAGE and transfer to PVDF. Membranes were incubated overnight with primary antibodies for furin (Enzo Life Sciences, Ann Arbor, MI) PCSK5, PCSK7, actin (Sigma, St. Louis, MO) and KISS1 at 4°C followed by with HRP-conjugated secondary antibody (room temperature; 1 hr). Signals were visualized using ECL (Pierce). For interaction studies of KISS1 and/or KP with PC, co-immunoprecipitation was done.

### shRNA transfection

shRNA plasmids targeting furin, PCSK5 and PCSK7 (Hush®, Origene, Rockville, MD) were transiently transfected using Lipofectamine 2000 (Life Technologies) using manufacture's protocol. Cells were co-transfected with 10∶1 shRNA per KISS1 cDNA molecule to maximize the probability of co-expression in the same cells. mRNA and protein of individual PC were analyzed after 72 hr by RT-PCR and immunoblot, respectively. CM was collected simultaneously and analyzed for KISS1 processing.

### Generation of mutant KISS1

*K57A, R67A and R124A* mutagenesis (hereafter the triple mutant is referred to as C1C2C3) was performed using QuikChange II XL (Stratagene, La Jolla, CA). The oligonucleotides were synthesized by Integrated DNA Technologies (Coralville, IA). Following mutagenesis, the sequence of the entire mutated cDNA was verified.

### Metastasis assays and animal studies

Melanoma cells (2×10^5^) were injected into the lateral tail veins of 3- to 4-wk-old female athymic mice (10 per group per experiment; Harlan Sprague-Dawley, Indianapolis, IN). Mice were housed at 23°C and given food and water *ad libitum*.

## Results

### KISS1 is secreted and processed into KP in multiple cell lines

**Figure 1:**
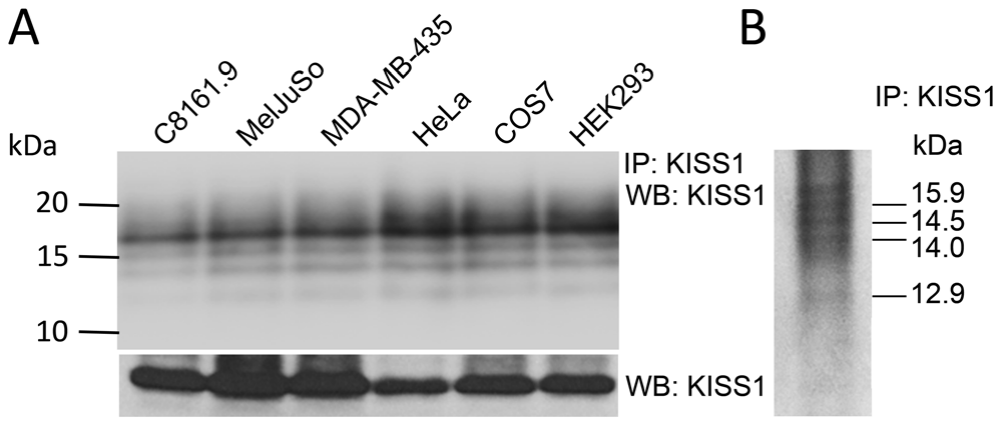
KISS1 is secreted and processed into kisspeptins (KP) in multiple cell lines. A. (*Top panel*) Conditioned media collected from cultures of C8161.9, MelJuSo, MDA-MB-435, HeLa, COS7 and HEK293 cell lines which exogenously express KISS1. KP were immunoprecipitated with a monoclonal antibody detecting KISS1 (6a4.27), separated by Tris-tricine SDS-PAGE and then immunoblotted with anti-KISS1 antibody (1∶1000). (*Bottom panel*) Full-length KISS1 was detected in whole cell lysates using anti-KISS1 antibody as above (Mr∼15.9 kDa; Note: lower molecular mass bands were not detected from whole cell lysates as previously published [15]). B. KISS1 processing into KP can be detected using immunoprecipitated KISS1/KP separated by Tris-tricine SDS-PAGE gel followed by Coomassie dye staining. KP bands were excised and analyzed by ESI-MS/MS [Figure S1]. KP sizes (approximate kDa) are predicted based upon the predicted PC cleavage sites in nascent KISS1 (See Figure 2A). KP sizes and sequence were confirmed by ESI-MS/MS and are shown schematically in Figure S1.

The presence of a secretion signal sequence in KISS1 was confirmed by our previous work in C8161.9 over-expressing KFM, an engineered version of KISS1 having an internal FLAG epitope [15]. Following secretion, KISS1 is processed into KP; however no KISS1 processing to KP was detected intracellularly in cell lysates [15]. To check whether this is a widely observed phenomenon, KISS1 was introduced into cell lines from different cellular origins and checked for secretion and extracellular processing (Figure 1A). All cell lines processed KISS1 to KP following secretion, suggesting a highly conserved mechanism to generate KP. KISS1 and kisspeptins were immunoprecipitated from conditioned medium (Figure 1B), excised and ESI-MS/MS confirmed that the secreted peptides were KISS1-derived (Figure S1). We emphasize that, since only one antibody was used, some processing variants would not be recognized. However, most of the processing variants predicted in the making of KP54 (Figure S1) will contain the epitope recognized by the antibody (since the antibody was generated by immunization with KP54 peptide). Some peptides are too small to readily visualize by immunoblot as well.

### PC are required for KP generation

**Figure 2:**
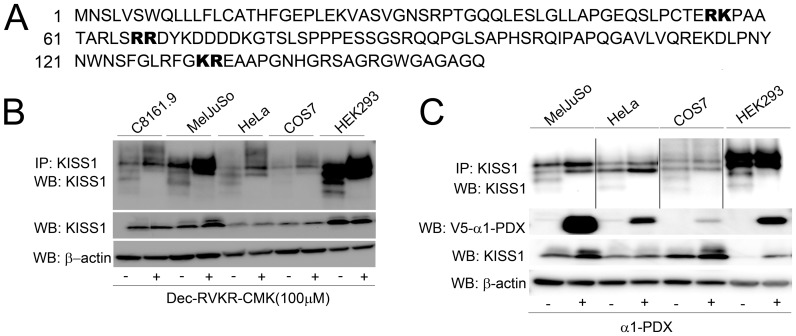
Proprotein convertases are required for KISS1 processing. A. Amino acid sequence of KISS1 showing three predicted PC cleavage sites − RK, RR and KR in bold. Note: DYKDDDDK at position 68 is the FLAG epitope tag used for several of the studies described in this manuscript. B. Treatment of C8161.9, MelJuSo, HeLa, COS7 and HEK293 cells over-expressing KISS1 with the general PC inhibitor Dec-RVKR-CMK (100 µM) blocked KISS1 processing as detected by immunoprecipitation and immunoblot using anti-KISS1 antibody [See Figure 1 and Materials and Methods for details]. C. Over-expression of KISS1 and the biological PC inhibitor V5-tagged α1-PDX also resulted in loss of KISS1 processing into KP. Polypeptide sizes correspond to those shown in Figure 1.

Analysis of KISS1 sequence showed three interspersed paired basic amino acid motifs (RK^57^, RR^67^, and KR^124^; Figure 2A). Since the FLAG-tagged version of KISS1 was used for most experiments, the sequence depicted in Figure 2A includes the FLAG epitope (DYKDDDDK) at amino acid position 68. Unless otherwise stated, amino acid positions in this manuscript refer to the native KISS1 molecule.

Hypothesizing that RK^57^, RR^67^, and KR^124^ dibasic motifs served as PC recognition sites, we used the general PC inhibitors Dec-RVKR-CMK and α1-PDX. CM collected from cells treated with both PC inhibitors showed complete abrogation of KISS1 processing (Figures 2B and 2C). Inhibition of KISS1 processing by α1-PDX, which more inhibits PC found in secretory pathways (PCSK1, PCSK2, furin, PCSK5, PCSK6 and PCSK7), refined our studies to those PC. Since furin, PCSK5 and PCSK7 mRNA were expressed consistently in all cell lines which process KISS1 (Table 1), subsequent studies focused on those three PC.

**Table 1:** RT-PCR analysis of the six members of PC family involved in the secretory pathway.

	Proprotein Convertases					
	PCSK1	PCSK2	Furin	PCSK5	PCSK6	PCSK7
**C8161.9**	**−**	**−**	**+**	**+**	**−**	**+**
**MelJuSo**	**+**	**+**	**+**	**+**	**+**	**+**
**MDA-MB-435**	**+**	**+**	**+**	**+**	**−**	**+**
**MDA-MB-231**	**+**	**−**	**+**	**+**	**+**	**+**
**HeLa**	**+**	**-**	**+**	**+**	**+**	**+**
**HEK293**	**+**	**+**	**+**	**+**	**+**	**+**

C8161.9, MelJuSo, MDA-MB-435, MDA-MB-231, HeLa and HEK293 cells that process KISS1 to KP showed consistent expression of furin, PCSK5 and PCSK7 in all of the cell lines. **+**  =  PC mRNA detected **−**  =  PC mRNA not detected. Note: This depiction is solely qualitative, not quantitative.

**Figure 3:**
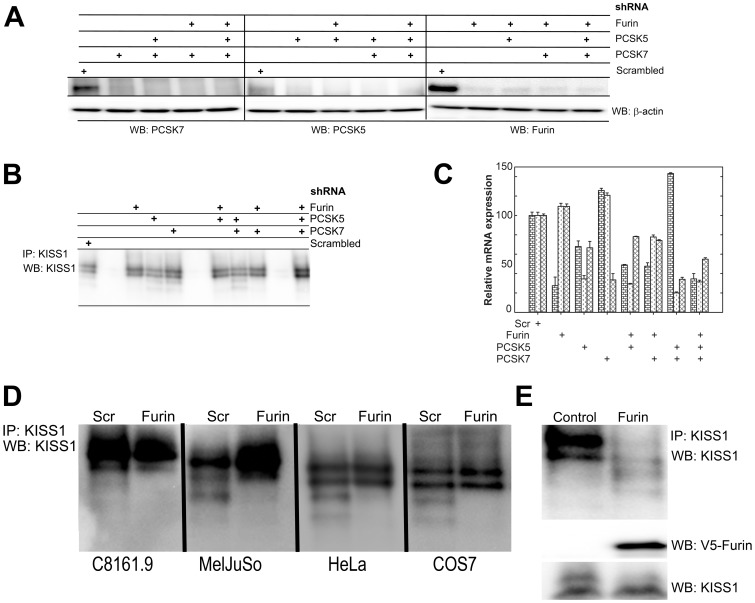
Furin is required for the generation of KP from KISS1. A, C. RT-PCR and immunoblot data showing reduced mRNA and protein levels of furin, PCSK5 and PCSK7 in HEK293 cells transduced with shRNA directed against each PC either individually or in combination. Bars in Panel C (by group; left to right)  =  furin, PCSK5 and PCSK7. B. shRNA-mediated knockdown of furin, PCSK5 and/or PCSK7 was in HEK293 cells over-expressing KISS1 show that cleavage of KISS1 is lost whenever furin is knocked down, but processing can still occur when PCSK5 or PCSK7 are knocked down. D. C8161.9, MelJuSo, HeLa and COS7 in which furin expression was knocked down by shRNA showed reduced or complete loss of KISS1 processing. KISS1 processing was observed in all cell lines when a scrambled control shRNA (Scr) was used. E. Restoration of furin activity in LoVo cells (which have no furin activity) by transduction of adenoviral particles with V5-tagged furin restored KISS1 processing to KP. LoVo cells transduced with an empty vector still do not show KISS1 cleavage into KP. Loading controls for V5-furin and KISS1 are shown using immunoblots with V5 and KISS1 antibodies. KISS1 and KP polypeptide sizes correspond to those shown in Figure 1.

*Furin is the PC involved in the generation of KP* - To identify the specific PC(s) involved in KISS1 processing, shRNA-mediated knockdown was used. shRNA targeting furin, PCSK5 and/or PCSK7 were transfected 10∶1 with KISS1 into HEK293 cells and assessed for knockdown (Figure 3A and 3C). PCSK5 and PCSK7 knockdown (∼25% of control expression at the mRNA level; <10% at the protein level) did not reduce KISS1 processing. Only shRNA targeting furin abrogated KISS1 –>KP processing (Figure 3B) and the knockdown efficiency was not cell line-dependent (Figure 3D). Note: shRNA-mediated knockdown of individual PC produced compensatory changes in the other PC; however, the changes were modest. Likewise, some of the shRNA reduced expression of other PC, but again the modifications were neither statistically significant nor consistent in every experiment. Importantly, whenever furin was lost, KISS1 processing was no longer observed.

LoVo human colorectal carcinoma cells are deficient in furin activity [26], [27]; therefore, they were used as a negative control for KISS1 processing by furin. Parental LoVo cells do not convert KISS1 into KP. However, transfection of active furin into LoVo cells restored KISS1 processing (Figure 3E), verifying that restoration of furin activity is sufficient for processing KISS1 to KP.

**Figure 4:**
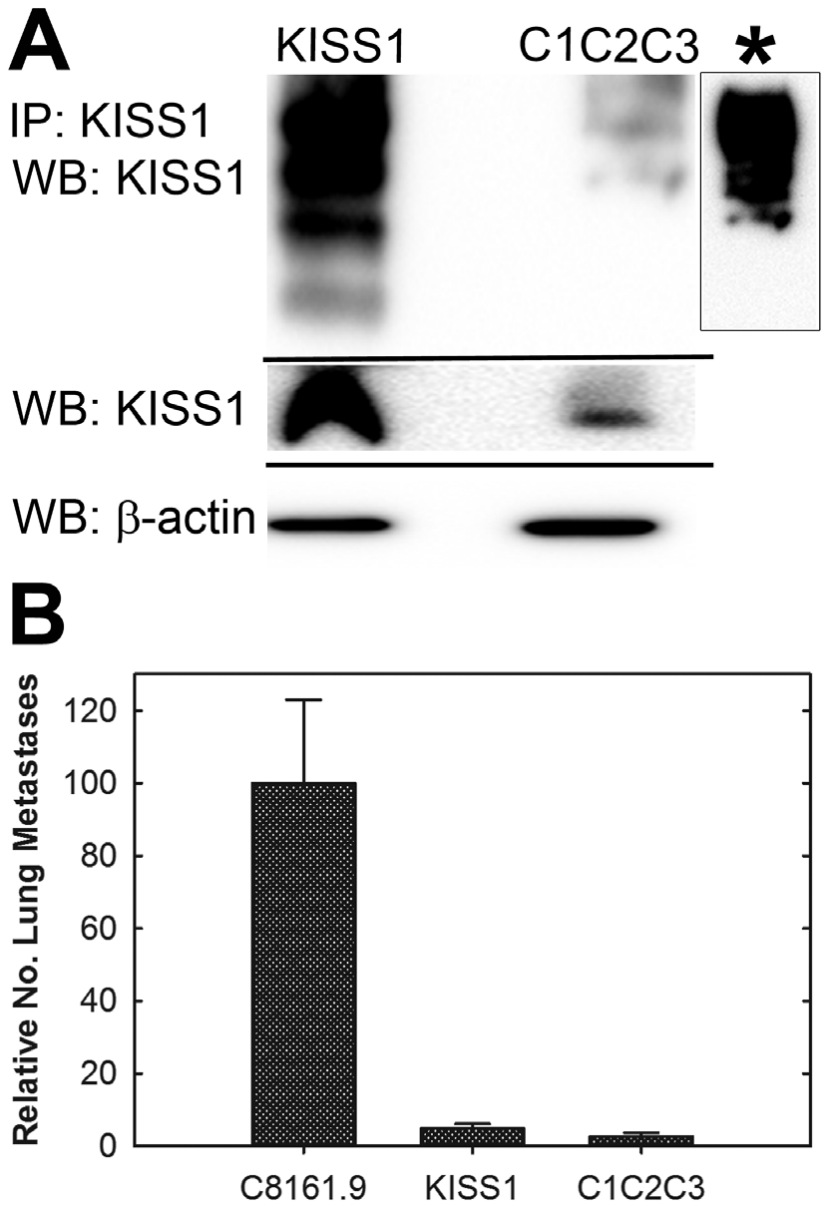
Mutation of putative PC sites in KISS1 blocks KP generation but does not abrogate metastasis suppressor function. A. Immunoblot showing KISS1 processing-deficient mutant (C1C2C3) which was mutated at the three putative furin processing sites (KISS1^K57A/R67A/R124A^). C1C2C3 was not processed into KP [Compare to Figure 1A and Figure 1B]. * indicates C1C2C3 lane which was over-exposed to highlight lack of laddering below the full-length KISS1 band. B. Relative lung metastases (mean ± SEM) compared to C8161.9 (647±149 metastases). KISS1 and C1C2C3 transfected cells were significantly less metastatic.

Since the above results implicate furin as the essential PC responsible for generation of KP from KISS1, all three putative furin processing sites were mutated and the metastasis suppressing capability of KISS1 was assessed. C8161.9 melanoma cells were transduced with KISS1 in which K^57^, R^67^ and R^124^ (C1C2C3) were mutated to alanine. KISS1 processing did not occur as confirmed by immunoblot (Figure 4A). In two independent experiments, C1C2C3 mutant-expressing C8161.9 cells were evaluated for lung colonization. While KISS1^WT^ blocked the formation of macroscopic metastasis as expected, C1C2C3-expressing cells were surprisingly metastasis suppressed at a level comparable to KISS1^WT^ (Figure 4B).

## Discussion

We reported previously that secretion is critical for the ability of KISS1 to suppress metastasis [15]. In that same report, we described the unexpected finding that KISS1 –>KP processing occurred outside of the tumor cells. And while the protein sequences of KP suggested that PC were responsible for proteolytic processing [10], [11], the identity of the responsible protease(s) has yet to be defined. In this report, our primary finding is that PC inhibition abrogates KISS1 processing. Moreover, furin was identified as the critical PC responsible for generation of KP. The data presented here show that other secretion-associated PC (i.e., PCSK5 and PCSK7) are not involved in KISS1 processing. However, we cannot preclude promiscuous processing in other cells or cell types or under different conditions.

Our data have implications regarding the functions of KISS1 as a secreted molecule. Since KISS1 that cannot be processed by furin still suppresses metastasis, one can infer that processing into KP is not as critical for metastasis suppression as secretion. Thus, cancer cells incapable of KISS1 processing should still be suppressed for metastasis as long as secretion is unabated.

The requirement of KP54 and KP10 in evoking KISS1R responses shows the importance of KISS1-KP processing in receptor-mediated signaling [6], [28]. KISS1R signaling is key to the hypothalamic-pituitary-gonadal axis, pubertal maturation, reproduction and fertility in mammals [2], [4], [29]–[31] as well as leptin feedback [32]–[34] and circadian responses [35], [36]. Thus, the discovery of furin as the enzyme required for generating KP places furin in a pivotal role of post-translationally regulating the function of KISS1 in multiple physiological conditions. Nonetheless, there are still remaining questions regarding KISS1 processing.

First, the lack of KISS1R expression in many tumor cell lines that can be metastasis suppressed by re-expression of KISS1 provided the first clue that autocrine signaling is not required for an anti-metastatic function [10]. When coupled with the data here, full-length KISS1 – possibly via a receptor-independent mechanism – could still suppress metastasis. The latter interpretation is supported by recent reports that KISS1 reverses aerobic glycolysis (Wen Liu *et al*. Submitted for publication). Alternatively, there may be as-yet-unidentified proteolytic cleavage of KISS1, a finding supported by data that KISS1 can be cleaved by MMP-9 [37].

Second, why, despite being found inside cells, does furin not process KISS1 intracellularly? Co-immunoprecipitation using KISS1 or furin antibodies demonstrated association outside, but not inside, cells (Figure S2). The findings imply involvement of other molecules (e.g., blocking inside or activating outside). Recent evidence that KISS1 can directly interact with PPAR-gamma co-activator 1α (Wen Liu, BH Beck, KS Vaidya, KT Nash, KP Feeley, SW Ballinger, KM Pounds, WL Denning, AR Diers, A Landar, A Dhar, T Iwakuma, DR Welch. The KISS1 metastasis suppressor appears to reverse the Warburg effect by enhancing mitochondrial biogenesis via a PGC1α pathway. Submitted for publication) lend credence to the hypothesis that interaction with other molecules could affect KP generation.

Third, throughout these studies, two bands were consistently detected in SDS-PAGE of conditioned medium (M_r_∼15.9 kDa and M_r_ ∼17 kDa), regardless whether cells were exposed to Dec-RVKR-CMK or shRNA targeting furin. KISS1 could be post-translationally modified or another protease (family) might cleave KISS1. If such modifications exist, they have the potential to impact proteolytic processing. We have not found reports in the literature regarding non-proteolytic post-translational modifications to date.

Now that furin has been identified as the critical enzyme responsible for KP generation, the field is opened to new targets to manipulate KISS1 in pathological conditions. By defining the role of furin in KISS1 processing, the opportunity to delineate roles of KISS1 processing ‘byproducts’ is more clearly defined.

## Supporting Information

Figure S1 **The processed peptides detected were derived from KISS1.** **A**. Predicted processing of KISS1 to KP leading to the generation of the final mature KP54 peptide. The size of KP detected in Figure 1 approximate the predicted sizes shown. (SS =  Signal Sequence, F =  FLAG tag, KP54 =  kisspeptin 54) **B**. Bands identified in Figure 1 Panel B were isolated and analyzed by ESI-MS/MS. Sequences identified were aligned with a full-length sequence of human KISS1. KP54 is highlighted by the underline. Confidence of sequence identity is depicted by black lines (99% confidence) or grey lines (>95% confidence) KP54 and other KP of smaller sizes are not detected because polyacrylamide gels are generally not suitable for detection of small polypeptides. **C**. The region of KISS1 consistently detected in all the peptide fragments derived from KISS1 has been shown and maps to the N-terminal region of KP54.
(TIF)

Figure S2 **Furin interacts with KISS1 only extracellularly.** Immunoblot showing co-immunoprecipitation of endogenous furin and over-expressed KISS1. Furin was immunoprecipitated from cell lysates (L) or conditioned media (CM) in MelJuSo and C8161.9 cells and probed for interaction using anti-KISS1 antibody. Reciprocal co-IP was also done. KISS1 and furin co-precipitate only in CM, but not intracellularly.
(TIF)
