# Supplementary figures and images for: Furin Is the Major Proprotein Convertase Required for KISS1-to-Kisspeptin Processing
Source: PLoS One. 2014 Jan 13;9(1):e84958. doi: 10.1371/journal.pone.0084958 (PMC3890299; doi:10.1371/journal.pone.0084958)

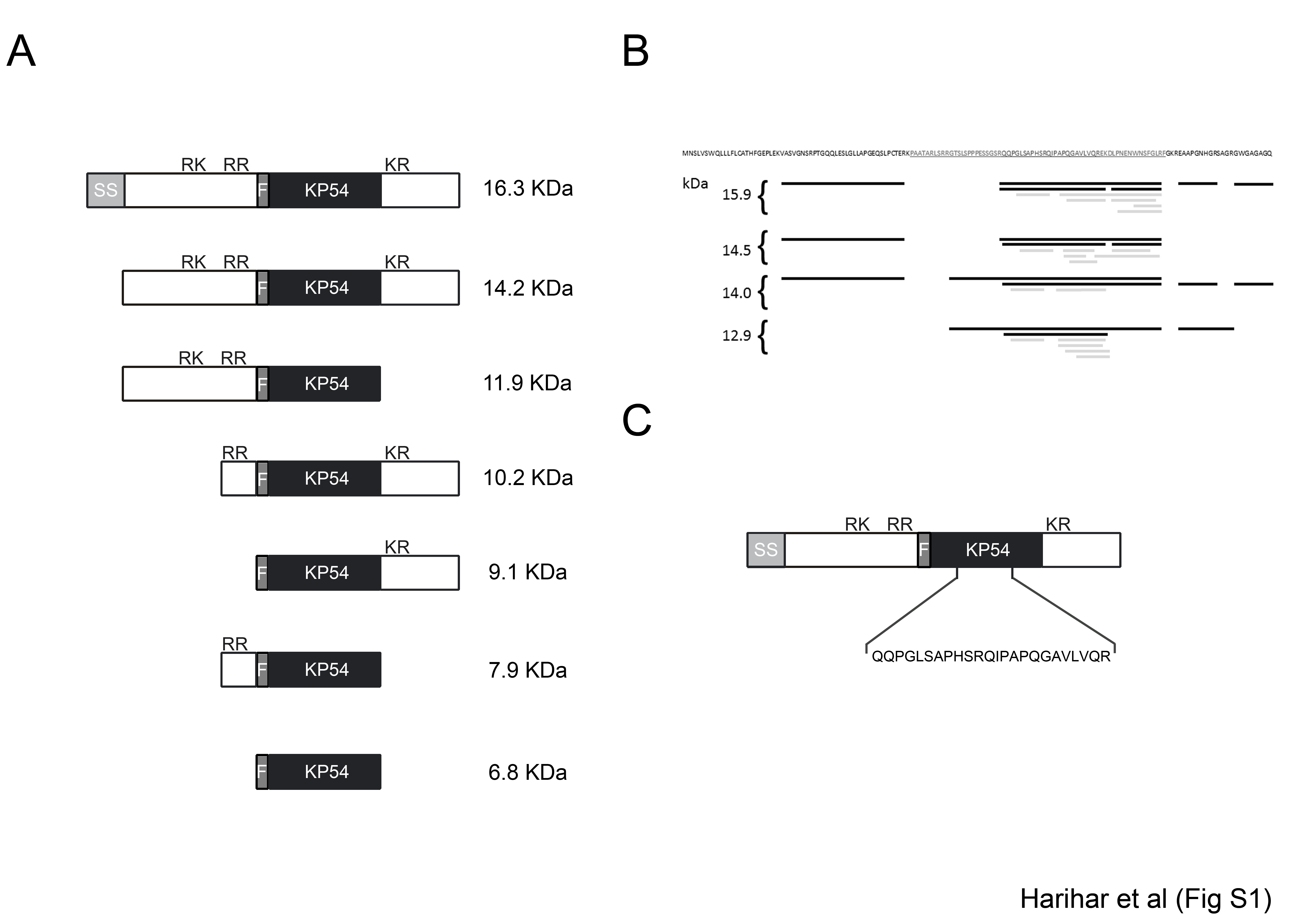

Supplement: Figure S1 — The processed peptides detected were derived from KISS1. A. Predicted processing of KISS1 to KP leading to the generation of the final mature KP54 peptide. The size of KP detected in Figure 1 approximate the predicted sizes shown. (SS = Signal Sequence, F = FLAG tag, KP54 = kisspeptin 54) B. Bands identified in Figure 1 Panel B were isolated and analyzed by ESI-MS/MS. Sequences identified were aligned with a full-length sequence of human KISS1. KP54 is highlighted by the underline. Confidence of sequence identity is depicted by black lines (99% confidence) or grey lines (>95% confidence) KP54 and other KP of smaller sizes are not detected because polyacrylamide gels are generally not suitable for detection of small polypeptides. C. The region of KISS1 consistently detected in all the peptide fragments derived from KISS1 has been shown and maps to the N-terminal region of KP54. (TIF) [file pone.0084958.s001.tif]

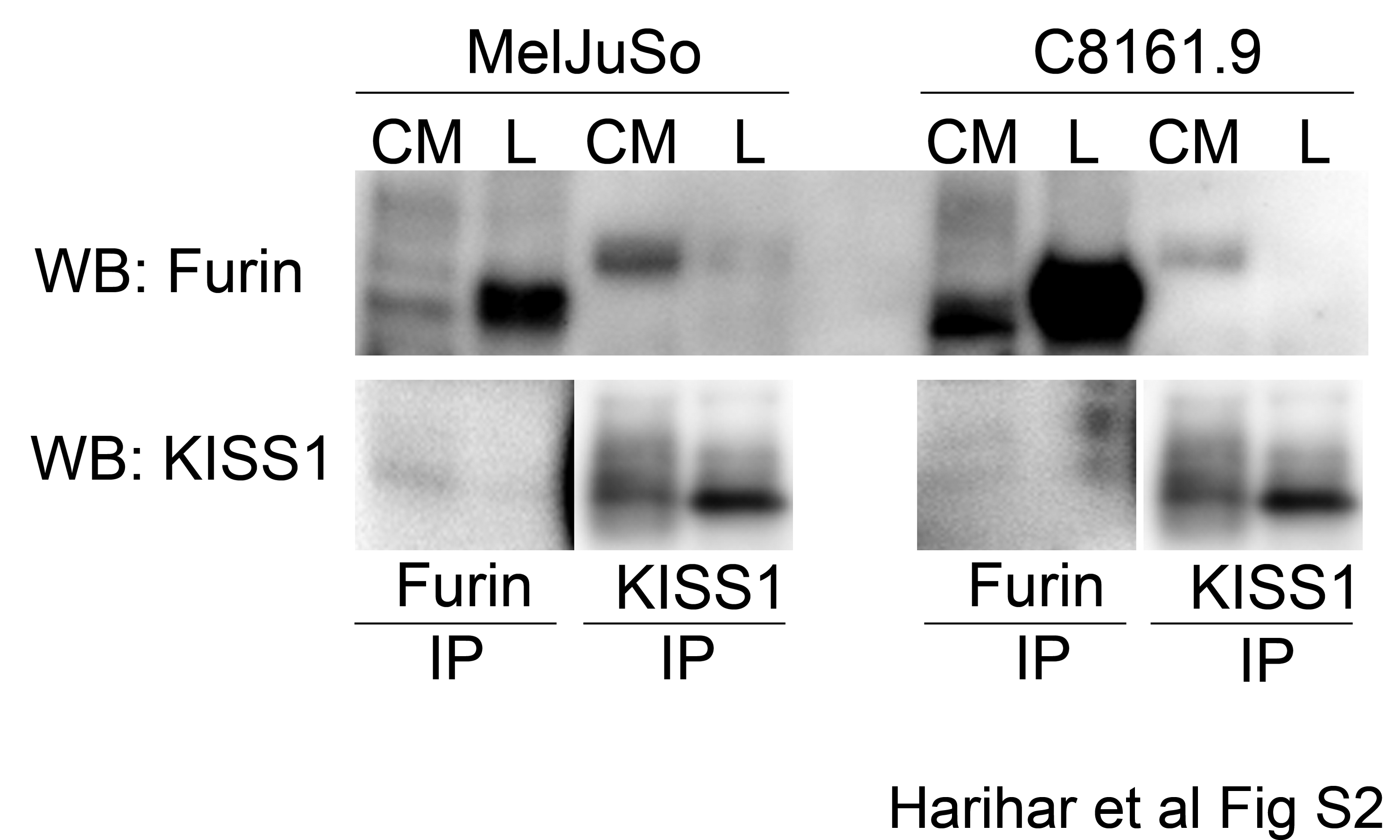

Supplement: Figure S2 — Furin interacts with KISS1 only extracellularly. Immunoblot showing co-immunoprecipitation of endogenous furin and over-expressed KISS1. Furin was immunoprecipitated from cell lysates (L) or conditioned media (CM) in MelJuSo and C8161.9 cells and probed for interaction using anti-KISS1 antibody. Reciprocal co-IP was also done. KISS1 and furin co-precipitate only in CM, but not intracellularly. (TIF) [file pone.0084958.s002.tif]
